# QTL mapping and haplotype analysis reveal candidate genes for seedling-stage salinity tolerance in rice

**DOI:** 10.3389/fpls.2026.1876706

**Published:** 2026-09-25

**Authors:** Wei Xu, Chunyan Ju, Xiaoding Ma, Bing Han, Xiaohong Guo, Zhengwu Zhao, Longzhi Han, Di Cui

**Affiliations:** 1 State Key Laboratory of Crop Gene Resources and Breeding, Institute of Crop Sciences, Chinese Academy of Agricultural Sciences, Beijing, China; 2 College of Agriculture, Heilongjiang Bayi Agricultural University, Daqing, China; 3 Chongqing Engineering Research Center of Specialty Crop Resources, College of Life Sciences, Chongqing Normal University, Chongqing, China

**Keywords:** candidate gene, haplotype analysis, linkage mapping, rice, salt stress

## Abstract

**Introduction:**

Soil salinization is a major constraint on rice production, and severe salinity levels lead to pronounced yield losses. Therefore, elucidating the genetic architecture of salinity tolerance and identifying candidate genes underlying this trait are crucial for molecular design breeding.

**Methods:**

In this study, a population of 253 recombinant inbred lines (RILs) derived from the salt-sensitive japonica cultivar Jileng 1 (JL1) and the salt-tolerant indica cultivar Milyang 23 (MY23) was evaluated for seedling-stage salinity tolerance under 0.9% NaCl stress.

**Results:**

Using multiple phenotypic indices and a high-density genetic map constructed from whole-genome resequencing data, we identified 43 QTLs associated with seedling-stage salinity tolerance, including 11 major-effect loci, each explaining more than 10% of the phenotypic variance. By integrating functional annotation, haplotype analysis, RNA-seq-based co-expression network analysis, and qRT-PCR validation, *OsFBX29* (*LOC_Os01g59910*) was prioritized as a strong candidate gene associated with seedling-stage salinity tolerance. Lines carrying the Hap2 haplotype exhibited enhanced salinity tolerance, and qRT-PCR analysis revealed significantly lower *OsFBX29* expression levels (*p* < 0.05) in Hap2-carrying lines after salt treatment, suggesting a potential association between reduced *OsFBX29* expression and enhanced salinity tolerance.

**Discussion:**

These findings provide new insights into the genetic architecture of seedling-stage salinity tolerance and offer potential genetic resources for breeding salinity-tolerant rice varieties.

## Introduction

Rice (*Oryza sativa* L.) is a major staple crop that is highly vulnerable to salinity stress, particularly during early developmental stages such as seedling establishment (Zeng and Shannon, 2000). Salinity stress generally refers to the excessive accumulation of soluble salts in soil or irrigation water, which imposes both osmotic stress and ionic toxicity on plants. In rice production systems, soil electrical conductivity (EC) above approximately 4 dS m^-^¹ can substantially restrict plant growth and productivity, and more severe salinity levels have been associated with pronounced yield losses (Zeng et al., 2001). At the physiological and biochemical levels, salinity stress inhibits water uptake and seedling establishment, disrupts Na^+^/K^+^ homeostasis, reduces chlorophyll accumulation and photosynthetic capacity, promotes reactive oxygen species (ROS) production, and induces membrane damage (Liu et al., 2022; Barman et al., 2025). Therefore, improving seedling-stage salinity tolerance in rice is essential for stable crop establishment in saline-alkaline soils and for maintaining yield stability. Although salinity tolerance at the reproductive stage is more directly associated with grain yield and remains an important target for salinity-tolerance breeding (Singh et al., 2021), seedling-stage tolerance is also critical because early salt injury can impair seedling survival, stand establishment, and subsequent crop performance in saline soils (Tiwari et al., 2022; Xu et al., 2023). Moreover, the seedling stage is a sensitive developmental phase during which genotypic differences in salinity responses can be effectively detected. Phenotyping at this stage under controlled hydroponic conditions also provides a rapid, reproducible, and scalable approach for screening large populations and identifying salinity-tolerance loci (Xu et al., 2023; Siddique et al., 2025). Therefore, the present study focused on seedling-stage salinity tolerance.

Salinity tolerance in rice is a complex quantitative trait governed by multiple genes. Previous quantitative trait locus (QTL) studies have identified numerous loci associated with salinity tolerance at both the seedling and reproductive stages, with many investigations focusing on seedling-stage responses (Singh et al., 2021; Geng et al., 2023; Xu et al., 2023). In linkage analyses using biparental populations, the major seedling-stage QTL Saltol was mapped to chromosome 1 in an IR29 × Pokkali RIL population and has since been widely used in breeding and functional validation studies (Krishnamurthy et al., 2020). In a panel of 308 F_4_ families derived from Sahel 317 and Madina Koyo, seedling-stage screening under salt stress was combined with construction of a high-density genetic map using 3,698 SNPs; composite interval mapping subsequently identified 13 QTLs associated with salt injury score (SIS), seedling height, shoot dry weight, and root length, distributed across chromosomes 2, 3, 4, 6, 7, 10, and 12 (Amoah et al., 2020). Using an F_2_ population derived from the salt-tolerant landrace Kalarata and the salt-sensitive cultivar Azucena, followed by seedling-stage phenotyping of F_3_ families, a linkage map based on 151 SSR and InDel markers was developed, and 13 QTLs were detected across 16 traits (de Ocampo et al., 2022). In addition, reciprocal mapping populations derived from the Bangladeshi salt-tolerant landrace Horkuch and the high-yielding cultivar IR29 were analyzed using an F_2:3_ design, and genetic maps constructed with DArTseq enabled the identification of 14 QTLs associated with salinity tolerance at the seedling and reproductive stages (Haque et al., 2022).

Despite the large number of salinity-tolerance QTLs mapped to date, only a limited number of loci have been resolved to causal genes by map-based cloning with clear mechanistic evidence. Among these, SKC1 was shown to encode the HKT-type sodium transporter *OsHKT1;5*, which regulates Na^^+^^ transport and maintains K^^+^^ homeostasis under salt stress, representing a landmark example of the transition from QTL to functional gene (Ren et al., 2005). Subsequent functional studies demonstrated that *OsHKT1;5* promotes Na^^+^^ retrieval from the xylem in vascular tissues of roots and stems, contributing to internal ion redistribution, restricting Na^^+^^ delivery to young leaves and reproductive organs, and thereby alleviating salt toxicity and enhancing salinity tolerance (Kobayashi et al., 2017). Similarly, *qSE3* was cloned and identified as the K^^+^^ transporter gene *OsHAK21*, which promotes seed germination and seedling establishment under salt stress (He et al., 2019). These cloned loci have revealed that ion transport, particularly Na^+^ transport, K^+^ uptake, and Na^+^/K^+^ homeostasis, represents an important mechanism underlying rice salinity tolerance.

However, for most reported salinity-tolerance QTLs, the specific molecular processes they regulate—such as Na^+^ exclusion, K^+^ uptake or retention, Na^+^/K^+^ homeostasis, osmotic adjustment, ROS detoxification, and stress-signaling pathways—remain incompletely understood, and many QTLs remain genetically broad and have not been sufficiently narrowed to specific candidate genes (Chen et al., 2021; Singh et al., 2021; Xu et al., 2023). In this study, we used the salt-sensitive japonica cultivar JL1 as the female parent and the salt-tolerant indica cultivar MY23 as the male parent to develop a recombinant inbred line (RIL) population comprising 253 F_10_ lines through continuous selfing using the single-seed descent method. Using a previously developed high-density genetic map based on whole-genome resequencing data, we performed QTL mapping for seedling-stage salinity tolerance and prioritized candidate genes associated with this trait. This study provides a foundation for elucidating the genetic basis of seedling-stage salinity tolerance in rice and offers valuable genetic resources for breeding salt-tolerant rice varieties.

## Materials and methods

### Plant materials and population development

The salt-sensitive japonica cultivar JL1 and the salt-tolerant indica cultivar MY23 were used as the female and male parents, respectively. The F_1_ plants and their progeny were continuously selfed using the single-seed descent method to develop a recombinant inbred line (RIL) population comprising 253 F_10_ lines. The RIL population was grown at the Changping Experimental Station of the Institute of Crop Sciences, Chinese Academy of Agricultural Sciences, under standard local agronomic management practices.

### Phenotype evaluation and statistical analysis

Seeds were dried at 50 °C for 2 days to break dormancy. For each line, 30 plump seeds were surface-sterilized with 1.4% sodium hypochlorite for 15 min and then rinsed three times with distilled water. Seeds were soaked at 35 °C for 24 h and subsequently germinated at 35 °C for a further 24 h. Twelve uniformly germinated seeds were selected and sown in 96-well plates, and seedlings were grown in a controlled growth chamber at 50% relative humidity under a 12 h light/12 h dark photoperiod (30 °C during the light period and 25 °C during the dark period). After 4 days of growth, seedlings were transferred to Yoshida nutrient solution, which was renewed every 2 days. At the two-leaf-one-heart stage, seedlings were transferred to Yoshida nutrient solution supplemented with 0.9% (w/v) NaCl (approximately 154 mM NaCl) to impose salinity stress, a concentration previously used for seedling-stage salinity-tolerance evaluation in rice (Zou et al., 2025; Theerakulpisut et al., 2016). The salt-treatment solution was renewed every 2 days to maintain stable treatment conditions throughout the experiment. Salinity injury was scored daily from 2 to 7 days after the onset of salt treatment, generating six time-point traits: 2dSST, 3dSST, 4dSST, 5dSST, 6dSST, and 7dSST, according to the 1–9 scale described by Han (2006), with lower scores indicating stronger salinity tolerance and higher scores indicating more severe salt injury. The single-plant salinity tolerance score (SST) was calculated as the average salinity-injury score of individual plants for each genotype. Chlorophyll content (CH) was measured using a SPAD meter. The DLR-derived salinity tolerance score (DSS) was assigned according to dead leaf rate thresholds: DSS = 1 for DLR ≤ 20%, 3 for 20% < DLR ≤ 40%, 5 for 40% < DLR ≤ 60%, 7 for 60% < DLR ≤ 80%, and 9 for 80% < DLR ≤ 100%. The SI-derived salinity tolerance score (ISS) was assigned according to salinity index thresholds: ISS = 1 for SI ≤ 15%, 3 for 15% < SI ≤ 30%, 5 for 30% < SI ≤ 60%, 7 for 60% < SI ≤ 85%, and 9 for 85% < SI ≤ 100%. All measurements were performed with three independent biological replicates.


$$\text{SSR}(\%)=\frac{\text{number of surviving seedlings}}{\text{total number of treated seedlings}}\times 100$$

$$\text{DLR}(\%)=\frac{\text{total number of dead leaves}}{\text{total number of leaves}}\times 100$$

$$\text{SI}(\%)=\frac{\sum (\text{number of plants at each injury grade}\times \text{corresponding grade value})}{\text{total number of plants}\times \text{maximum grade value}}\times 100$$

### Linkage map construction and QTL detection

A high-density genetic linkage map based on whole-genome resequencing data had been developed for this RIL population in our previous study (Geng et al., 2023). The previously developed map comprised 2,921 bin markers spanning 1,408.03 cM, with an average genetic length of 117.34 cM per chromosome. The average inter-marker genetic distance was 0.48 cM. QTL detection in this study was performed using the BIP module in QTL IciMapping (Meng et al., 2015). Permutation tests with 1,000 permutations were conducted separately for each phenotypic trait. The empirical LOD thresholds were all ≤ 2.5 across traits; therefore, a common threshold of LOD ≥ 2.5 was used to retain QTLs for further analysis. To reduce false-positive results, QTLs detected repeatedly across related traits or adjacent time points, especially those with relatively high LOD scores and/or PVE values, were interpreted with greater confidence and were prioritized for candidate-gene discovery, whereas isolated trait-specific QTLs were treated as putative loci requiring further validation. Additive effects and the proportion of phenotypic variance explained (PVE) by each QTL were subsequently estimated. QTL nomenclature followed the guidelines of McCouch et al. (1997). The linkage map was visualized using the LinkageMapView package in R (Ouellette et al., 2018).

### SNP annotation and haplotype analysis

Using the IRGSP-1.0 japonica reference genome, SNPs were subjected to quality control according to the genotyping and marker-filtering criteria. Sites with missing parental genotypes were excluded, and SNPs with parental base support ≥5 and progeny base support ≥3 were retained for downstream analyses. A total of 2,822,607 SNPs were annotated using SnpEff (Cingolani et al., 2012). Nonsynonymous SNPs within the coding sequences and polymorphic SNPs within the 2-kb upstream promoter regions of candidate genes were included in site-wise haplotype analyses. At each retained SNP site, RILs carrying the two parental homozygous genotypes were assigned to Hap1 and Hap2, respectively (Geng et al., 2023), whereas lines with heterozygous or missing genotypes were excluded. Phenotypic differences between Hap1 and Hap2 at each site were evaluated using Welch’s *t*-test.

### RNA-seq and qRT-PCR validation

A representative salt-tolerant line and a salt-sensitive line were selected from the RIL population. Leaf samples were collected from these lines at two time points: 0 h (before treatment) and 6 h after the onset of 0.9% NaCl treatment, with three biological replicates per line per time point. Samples were immediately snap-frozen in liquid nitrogen and stored at −80 °C until RNA extraction. Total RNA was extracted using an RNA extraction kit (Novogene, Beijing, China). Poly(A)-enriched libraries were constructed and sequenced on an Illumina HiSeq 2500 platform. Raw reads were quality-filtered using fastp (v0.20.0) (Chen et al., 2018) and aligned to the IRGSP-1.0 rice reference genome using STAR (v2.7.9a) (Dobin et al., 2013), with gene models annotated according to MSU v7.0. Gene-level read counts were quantified using featureCounts (Liao et al., 2014), and differential expression analysis was performed using DESeq2 (Love et al., 2014). Genes with an adjusted *p*-value < 0.05 and |log_2_FC| ≥ 1 were defined as differentially expressed genes. Weighted gene co-expression network analysis (WGCNA) was conducted using normalized expression data from the representative salt-tolerant and salt-sensitive lines to identify gene modules associated with the salt-stress response. Modules significantly correlated with treatment conditions (|r| ≥ 0.90, *p* < 0.001) were selected for further analysis. To account for potential variation among representative lines, module–condition associations were interpreted as group-level co-expression patterns and were used only as supporting evidence for candidate-gene prioritization. Network visualization was performed in Cytoscape (v3.7.1) (Shannon et al., 2003). To validate the RNA-seq results, quantitative reverse-transcription PCR (qRT-PCR) was performed using four representative salt-tolerant lines and four representative salt-sensitive lines. Leaf samples were collected at 0 h and 6 h after the onset of 0.9% NaCl treatment. qRT-PCR was conducted using THUNDERBIRD SYBR qPCR Mix (Toyobo, Osaka, Japan), with primer sequences provided in **Supplementary Table S1**. Each sample was assayed in three technical replicates across three biological replicates. The ubiquitin gene (*UBI*) was used as the internal reference. Relative transcript levels were calculated using the 2^^−ΔΔCt^ method (Livak and Schmittgen, 2001), and statistical significance was determined using Student’s t-test in R (v4.2.2). Before the t-test, the normality of residuals was assessed, and no significant deviation from normality was detected. Because only one pre-specified pairwise comparison between the treatment group and the corresponding control group was performed for each target gene, multiple-comparison correction was not applied.

## Results

### Assessment of seedling-stage salinity tolerance

After 7 days of salt treatment, MY23 seedlings showed markedly higher vigor and better growth than JL1 (**Figure 1A**). Consistent with this observation, salinity tolerance scores of the two parents clearly diverged from day 2 to day 7: scores for JL1 and MY23 were 5.67 and 2.33, 7.00 and 3.67, 9.00 and 3.67, 9.00 and 4.33, 9.00 and 4.33, and 9.00 and 4.33, respectively (**Figure 1B**; **Table 1**). For the female parent JL1, the values of seedling survival rate (SSR), chlorophyll content (CH), dead leaf rate (DLR), DLR-derived salinity tolerance score (DSS), salinity index (SI), SI-derived salinity tolerance score (ISS), and single-plant salinity tolerance score (SST) were 16.67%, 0.99, 84.44%, 8.33, 96.30%, 9.00, and 8.67, respectively; the corresponding values for the male parent MY23 were 69.44%, 22.06, 36.03%, 3.67, 64.81%, 6.33, and 5.83, respectively. Collectively, these results confirmed that MY23 exhibited substantially greater salinity tolerance than JL1.

**Figure 1:**
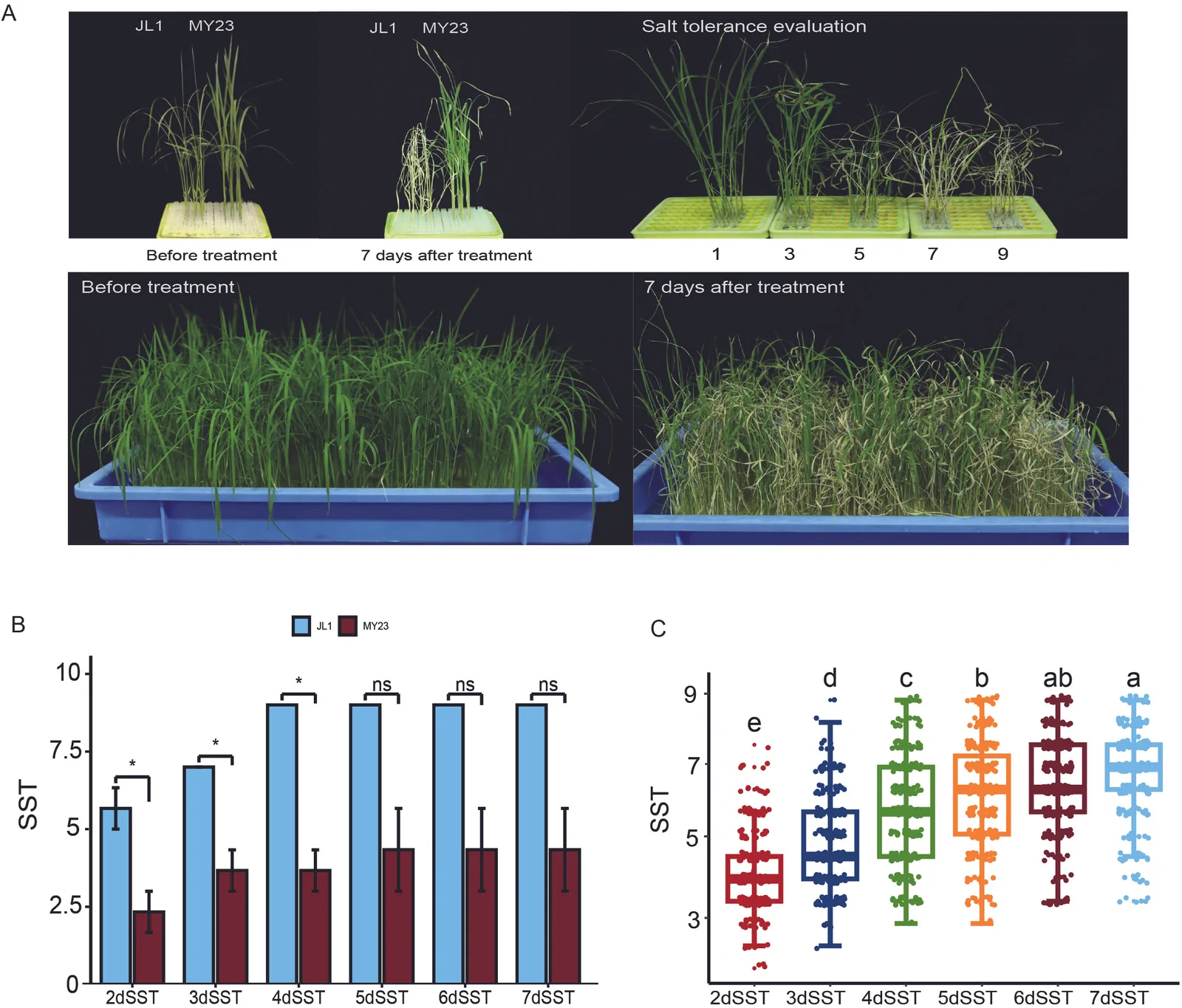
Seedling-stage salinity tolerance of the parental lines and the RIL population. **(A)** Phenotypes of the two parents and representative lines before and after 7 days of salt treatment, together with single-plant salinity tolerance scores (SST). **(B)** Salinity tolerance scores from day 2 to day 7 after salt treatment (2dSST–7dSST) for JL1 and MY23. Values are presented as mean ± SD of three independent biological replicates, with 12 seedlings per replicate (n = 36 plants per phenotype). Error bars represent SD; error bars are not visible when SD = 0. Differences between JL1 and MY23 at each time point were evaluated using Welch’s t-test. *indicates a significant difference at *p* < 0.05, whereas ns indicates no significant difference. **(C)** Distribution of salinity tolerance scores from day 2 to day 7 after salt treatment (2dSST–7dSST) across the RIL population; different letters indicate statistically significant differences based on multiple comparisons (*p* < 0.05).

**Table 1:** Statistical analysis of salt tolerance traits in RIL population at the seedling stage.

Trait	Parent		RIL				
	JL1	MY23	Mean ± SD	CV%	Range	Skewness	Kurtosis
2dSST	5.67	2.33	3.76 ± 1.20	31.80%	1.00-7.67	0.74	0.51
3dSST	7.00	3.67	4.64 ± 1.43	30.90%	1.67-9.00	0.47	-0.52
4dSST	9.00	3.67	5.65 ± 1.63	28.83%	2.33-9.00	0.04	-1.01
5dSST	9.00	4.33	6.16 ± 1.59	25.81%	2.33-9.00	-0.27	-0.68
6dSST	9.00	4.33	6.43 ± 1.45	22.58%	3.00-9.00	-0.52	-0.12
7dSST	9.00	4.33	6.77 ± 1.32	19.57%	3.00-9.00	-0.91	0.66
SSR	16.67%	69.44%	58.71% ± 25.85%	44.03%	2.78-100.00%	-0.34	-0.91
CH	0.99	22.06	5.94 ± 5.03	84.70%	0.00-27.91	1.57	2.67
DLR	84.44%	36.03%	71.24% ± 18.80%	26.39%	15.56-100.00%	-0.67	0.01
DSS	8.33	3.67	6.78 ± 1.82	26.89%	1.67-9.00	-0.82	0.11
SI	96.30%	64.81%	83.44% ± 9.96%	11.94%	40.74-99.38%	-1.05	1.68
ISS	9.00	6.33	7.91 ± 0.92	11.57%	4.33-9.00	-0.82	0.75
SST	8.67	5.83	7.31 ± 1.06	14.46%	3.50-8.94	-0.94	0.85

In the RIL population, mean salinity tolerance scores from day 2 to day 7 were 3.76, 4.64, 5.65, 6.16, 6.43, and 6.77, respectively (**Figure 1C**; **Table 1**). Mean values of SSR, CH, DLR, DSS, SI, ISS, and SST were 58.71%, 5.94, 71.24%, 6.78, 83.44%, 7.91, and 7.31, respectively. These salinity-related traits exhibited substantial phenotypic variation across the population, with coefficients of variation ranging from 11.57% to 84.70%, reflecting rich genetic diversity among the lines (**Supplementary Figure S1**). Further correlation analysis was performed among the salinity-related traits in the RIL population (**Supplementary Figure S2**). DLR, DSS, SI, ISS, and SST were significantly positively correlated with each other (r = 0.79–0.98, *P* < 0.01), with the highest correlation observed between DLR and DSS (r = 0.98, *P* < 0.01). Similarly, salinity tolerance scores at different evaluation stages (2dSST–7dSST) exhibited highly significant positive correlations (r = 0.60–0.96, *P* < 0.01). Conversely, SSR and CH exhibited significant negative correlations with DLR, DSS, SI, ISS, SST, and 2dSST–7dSST, with correlation coefficients ranging from −0.53 to −0.90 (*P* < 0.01).

### QTL mapping of salinity tolerance

Using a high-density linkage map developed in our previous study (Geng et al., 2023), QTL mapping was performed based on multiple seedling-stage salinity-related indices, including 2dSST to 7dSST, SSR, CH, DLR, DSS, SI, ISS, and SST (**Table 2**; **Figure 2**). In total, 43 QTLs were detected on chromosomes 1, 2, 3, 9, 10, and 12, with LOD scores ranging from 2.53 to 47.99 and phenotypic variance explained (PVE) ranging from 0.69% to 19.78%. Of these, 11 major QTLs each showed PVE > 10%.

**Table 2:** QTLs detected for salt tolerance in the RIL population at the seedling stage.

Trait	QTL	Chr	Physical interval (Mb)	LOD	PVE (%)	Add	Reported QTL(s)	Reference(s)
2dSST	*q2dSST1^+^*	1	2.11–2.34	11.61	14.37	0.45		
	*q2dSST3*	3	1.33–1.53	4.46	5.19	0.27		
	*q2dSST9*	9	9.80–12.23	9.57	12.08	0.41	RRDW-associated GWAS locus (lead SNP Chr9: 9.825 Mb)	Xu et al. (2024)
	*q2dSST10*	10	15.74–15.92	3.30	3.79	-0.23	*SalTol10-1*	Islam et al. (2011)
	*q2dSST12*	12	1.42–1.73	4.89	5.48	0.36		
3dSST	*q3dSST1-1^+^*	1	25.44–25.82	3.27	4.02	0.28		
	*q3dSST1-2^+^*	1	2.11–2.34	6.95	9.16	0.42		
	*q3dSST9*	9	9.80–12.23	12.26	17.86	0.58	RRDW-associated GWAS locus (lead SNP Chr9: 9.825 Mb)	Xu et al. (2024)
4dSST	*q4dSST1-1*	*1*	23.34–25.11	4.13	5.37	0.39	*qSNC1*	Naveed et al. (2018)
	*q4dSST1-2*	1	1.24–1.43	5.44	5.99	0.40		
	*q4dSST9+*	9	9.98–10.34	12.10	14.07	0.62		
	*q4dSST10^+^*	10	12.93–13.14	8.68	9.94	-0.54	*SalTol10-1*; *qSKC10*	Islam et al. (2011); Qiu et al. (2015)
5dSST	*q5dSST1-1*	1	23.34–25.11	5.86	8.60	0.45	*qSNC1*	Naveed et al. (2018)
	*q5dSST1-2*	1	1.12–1.33	2.91	3.59	0.29		
	*q5dSST9^+^*	9	9.98–10.34	10.78	14.19	0.57		
	*q5dSST10^+^*	10	13.72–13.94	6.23	8.18	-0.44	*SalTol10-1*	Islam et al. (2011)
6dSST	*q6dSST1-1*	1	23.34–25.11	5.90	7.68	0.41	*qSNC1*	Naveed et al. (2018)
	*q6dSST1-2*	1	1.04–1.24	3.24	3.63	0.28		
	*q6dSST2*	2	9.03–9.16	3.51	3.99	-0.29		
	*q6dSST9^+^*	9	9.98–10.34	9.43	10.93	0.48		
	*q6dSST10^+^*	10	13.72–13.94	6.73	7.87	-0.41	*SalTol10-1*	Islam et al. (2011)
7dSST	*q7dSST1*	1	23.34–25.11	4.69	1.84	0.36	*qSNC1*	Naveed et al. (2018)
	*q7dSST9^+^*	9	9.98–10.34	40.57	19.14	1.14		
	*q7dSST10*	10	12.93–13.14	3.09	1.02	-0.27	*SalTol10-1; qSKC10*	Islam et al. (2011); Qiu et al. (2015)
SSR	*qSSR1^+^*	1	2.11–2.34	5.89	8.30	-0.07		
	*qSSR9^+^*	9	9.98–10.34	11.05	16.10	-0.10		
CH	*qCH1^+^*	1	35.23–35.44	4.52	5.73	-1.43	*qPH1.1s; QSkc1*	Mohammadi et al. (2013); Cheng et al. (2012)
	*qCH9^+^*	9	9.98–10.34	7.25	9.43	-1.64		
	*qCH10*	10	12.93–13.14	4.37	5.56	1.30	*SalTol10-1*; *qSKC10*	Islam et al. (2011); Qiu et al. (2015)
DLR	*qDLR1*	1	23.34–25.11	3.85	6.84	0.05	*qSNC1*	Naveed et al. (2018)
	*qDLR9^+^*	9	9.98–10.34	5.53	8.55	0.05		
	*qDLR10^+^*	10	13.72–13.94	5.63	8.76	-0.05	*SalTol10-1*	Islam et al. (2011)
DSS	*qDSS1*	1	23.34–25.11	4.15	7.11	0.48	*qSNC1*	Naveed et al. (2018)
	*qDSS9^+^*	9	9.98–10.34	5.21	7.71	0.49		
	*qDSS10^+^*	10	12.93–13.14	5.54	8.30	-0.53	*SalTol10-1*; *qSKC10*	Islam et al. (2011); Qiu et al. (2015)
SI	*qSI3*	3	4.14–4.28	2.85	4.90	0.02		
	*qSI9^+^*	9	9.98–10.34	7.17	12.76	0.03		
ISS	*qISS1^+^*	1	25.44–25.82	2.73	0.72	0.18		
	*qISS9^+^*	9	9.98–10.34	47.99	19.78	0.95		
	*qISS10^+^*	10	13.72–13.94	2.53	0.69	-0.18	*SalTol10-1*	Islam et al. (2011)
SST	*qSST1^+^*	1	35.23–35.44	2.62	3.81	0.22	*qPH1.1s; QSkc1*	Mohammadi et al. (2013); Cheng et al. (2012)
	*qSST9^+^*	9	9.98–10.34	8.83	13.50	0.38		
	*qSST10^+^*	10	12.93–13.14	3.92	5.79	-0.25	*SalTol10-1*; *qSKC10*	Islam et al. (2011); Qiu et al. (2015)

Chr, chromosome; PVE, phenotypic variation explained; Add, additive effect; positive values indicate that the JL1 allele increases the phenotypic value, whereas negative values indicate that the MY23 allele increases the phenotypic value. For SST-related traits, increased phenotypic values indicate increased salt injury rather than enhanced salt tolerance. ^+^indicates prioritized QTLs.

**Figure 2:**
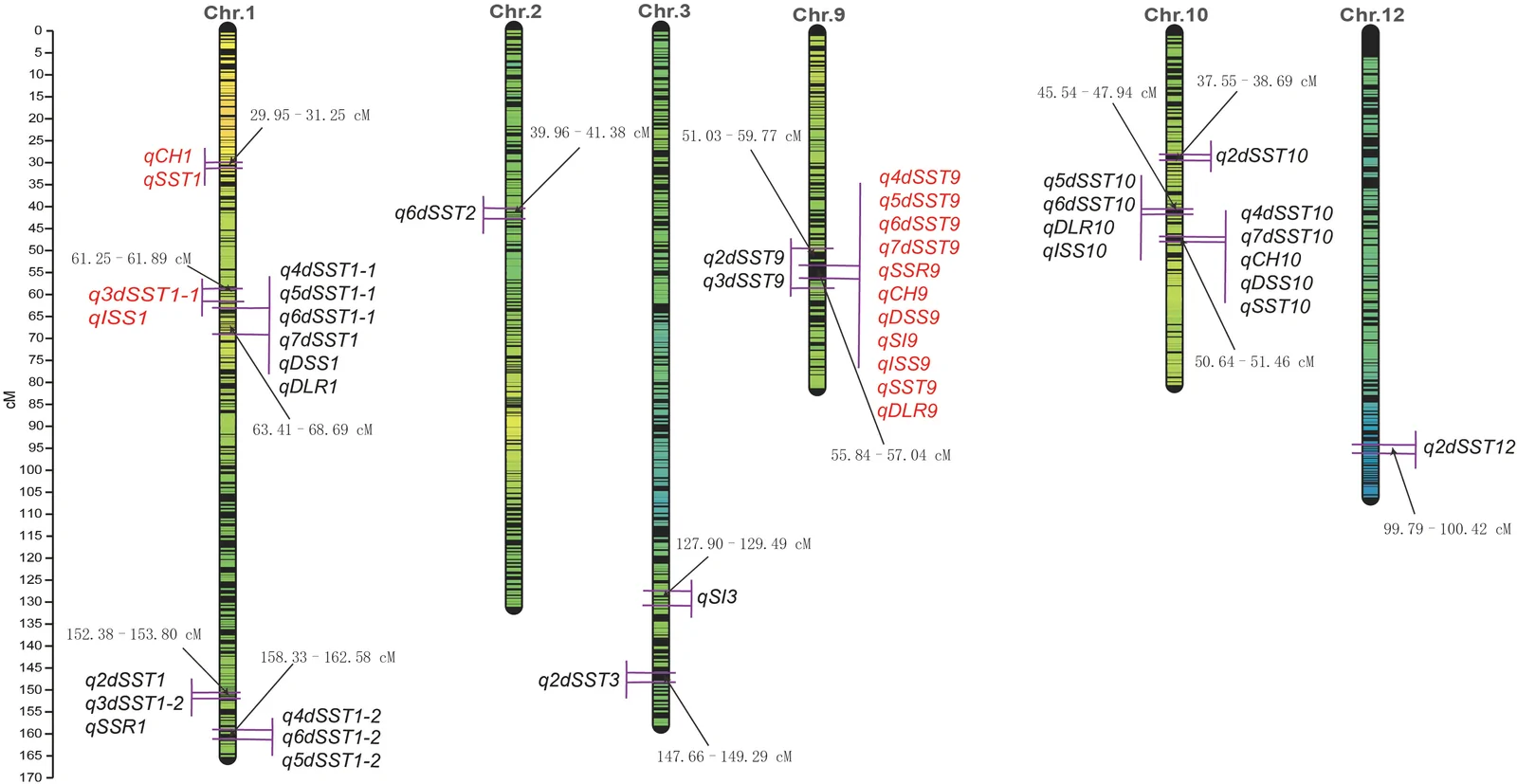
Chromosomal distribution of seedling-stage salinity-tolerance QTLs in the RIL population. A total of 43 QTLs are distributed across chromosomes 1, 2, 3, 9, 10, and 12. Purple bars indicate QTL intervals projected onto the linkage map according to the physical and genetic positions of the corresponding bin markers, and boxed values indicate the corresponding genetic intervals in cM. Red labels highlight QTL regions on chromosomes 1 and 9. The color gradient within each chromosome represents marker density along the linkage map. Genetic distance is indicated in cM.

Five QTLs were associated with 2dSST, among which *q2dSST1* and *q2dSST9* explained 14.37% and 12.08% of the phenotypic variance, respectively. For 3dSST, three QTLs were identified, with the highest contribution from *q3dSST9* at 17.86%. Four QTLs were associated with 4dSST and 5dSST, with *q4dSST9* and *q5dSST9* contributing the most at 14.07% and 14.19%, respectively. Five QTLs were associated with 6dSST, with *q6dSST9* contributing the most at 10.93%. Three QTLs were linked to 7dSST, with *q7dSST9* showing the highest contribution at 19.14%. Two QTLs were associated with SSR, with *qSSR1* and *qSSR9* explaining 8.30% and 16.10% of the phenotypic variance, respectively. Three QTLs were associated with each of CH, DLR, DSS, and SST, with *qSST9* showing the highest contribution at 13.50%. Two QTLs were linked to SI, and three to ISS, with *qISS9* contributing the most at 19.78%.

Overlapping QTL intervals were observed among several salinity-related traits, including traits measured at adjacent time points. These overlapping intervals were referred to as co-localized or positionally clustered QTL regions for candidate-region prioritization. As interval overlap alone does not necessarily indicate a shared causal locus, these co-localized or positionally clustered regions were used only for candidate-gene prioritization based on repeated detection, LOD scores, and PVE values. On chromosome 1, the QTL clusters were arranged from top to bottom on the linkage map as follows: *qCH1* and *qSST1*; *q3dSST1–1* and *qISS1*; *q4dSST1-1*, *q5dSST1-1*, *q6dSST1-1*, *q7dSST1*, *qDLR1*, and *qDSS1*; *q2dSST1*, *q3dSST1-2*, and *qSSR1*; and *q4dSST1-2*, *q5dSST1-2*, and *q6dSST1-2*. On chromosome 9, two positionally clustered QTL regions were identified: *q2dSST9* and *q3dSST9*; and *q4dSST9*, *q5dSST9*, *q6dSST9*, *q7dSST9*, *qSSR9*, *qCH9*, *qDLR9*, *qDSS9*, *qSI9*, *qISS9*, and *qSST9*. On chromosome 10, the co-localized QTL clusters were arranged from top to bottom as follows: *q5dSST10*, *q6dSST10*, *qDLR10*, and *qISS10*; and *q4dSST10*, *q7dSST10*, *qCH10*, *qDSS10*, and *qSST10*. Based on their repeated detection across related traits or traits measured at adjacent time points, together with relatively high LOD scores and PVE values, QTL regions on chromosomes 1, 9, and 10 were prioritized for subsequent candidate-gene discovery and haplotype analysis (**Figure 2**).

### Haplotype analysis of candidate genes

More than 300 genes were identified within the three major QTL intervals on chromosomes 1, 9, and 10 (**Supplementary Table S2**), including 43 homologs of genes previously reported to be involved in salinity tolerance or salt stress responses. Of these 43 genes, seven harbored nonsynonymous SNPs based on SnpEff annotation. Based on nonsynonymous SNP screening and functional annotation, four candidate genes potentially associated with salinity tolerance were prioritized. Among these, *OsFBX29* (*LOC_Os01g59910*) and *OsFBX339* (*LOC_Os09g35680*) encode F-box domain-containing proteins. *OsMYB21* (*LOC_Os01g63460*) encodes a putative MYB-family transcription factor, whereas *OsSub59* (*LOC_Os09g36110*) encodes a putative subtilisin homolog.

SNPs located in the coding sequences and 2-kb promoter regions of the four candidate genes were identified by comparing JL1 and MY23. For haplotype analysis, RILs carrying the homozygous JL1-derived genotype were classified as Hap1, whereas those carrying the homozygous MY23-derived genotype were classified as Hap2. For example, based on nonsynonymous variation in the CDS, Hap1 and Hap2 of *OsFBX29* were represented by T (n = 174) and C (n = 72), respectively; those of *OsMYB21* were represented by T (n = 196) and C (n = 53); those of *OsFBX339* were represented by A (n = 161) and G (n = 87); and those of *OsSub59* were represented by GGCCGGCA (n = 111) and GGCCGATC (n = 50), respectively (**Table 3**).

**Table 3:** Haplotype information for the four candidate genes based on nonsynonymous variations in CDS.

Gene	Functional annotation	Hap1/no.	Hap2/no.
*LOC_Os01g59910*	*OsFBX29*, F-box domain-containing protein	T/174	C/72
*LOC_Os01g63460*	*OsMYB21*, putative MYB-family transcription factor	T/196	C/53
*LOC_Os09g35680*	*OsFBX339*, F-box domain-containing protein	A/161	G/87
*LOC_Os09g36110*	*OsSub59*, putative subtilisin homolog	GGCCGGCA/111	GGCCGATC/50

No., number of RILs. Hap1 and Hap2 represent the JL1-derived and MY23-derived homozygous haplotypes, respectively. Lines with heterozygous or missing genotypes and other unclassified/non-parental haplotypes were excluded from the haplotype comparison.

For *OsFBX29*, one nonsynonymous SNP was identified in the coding region at Chr1:34,645,688 (T>C), together with 18 SNPs in the 2-kb promoter region spanning Chr1:34,646,430 to Chr1:34,648,040 (**Figure 3**). Based on significance tests of 6dSST between the two haplotypes defined by each SNP, 12 SNPs showed significant differences (*P* < 0.05; **Figure 3**). Similarly, significant differences in 6dSST were also detected between the two haplotypes defined by respective SNPs in *OsFBX339*, *OsMYB21*, and *OsSub59* (*P* < 0.05; **Supplementary Figures S3**-**S5**).

**Figure 3:**
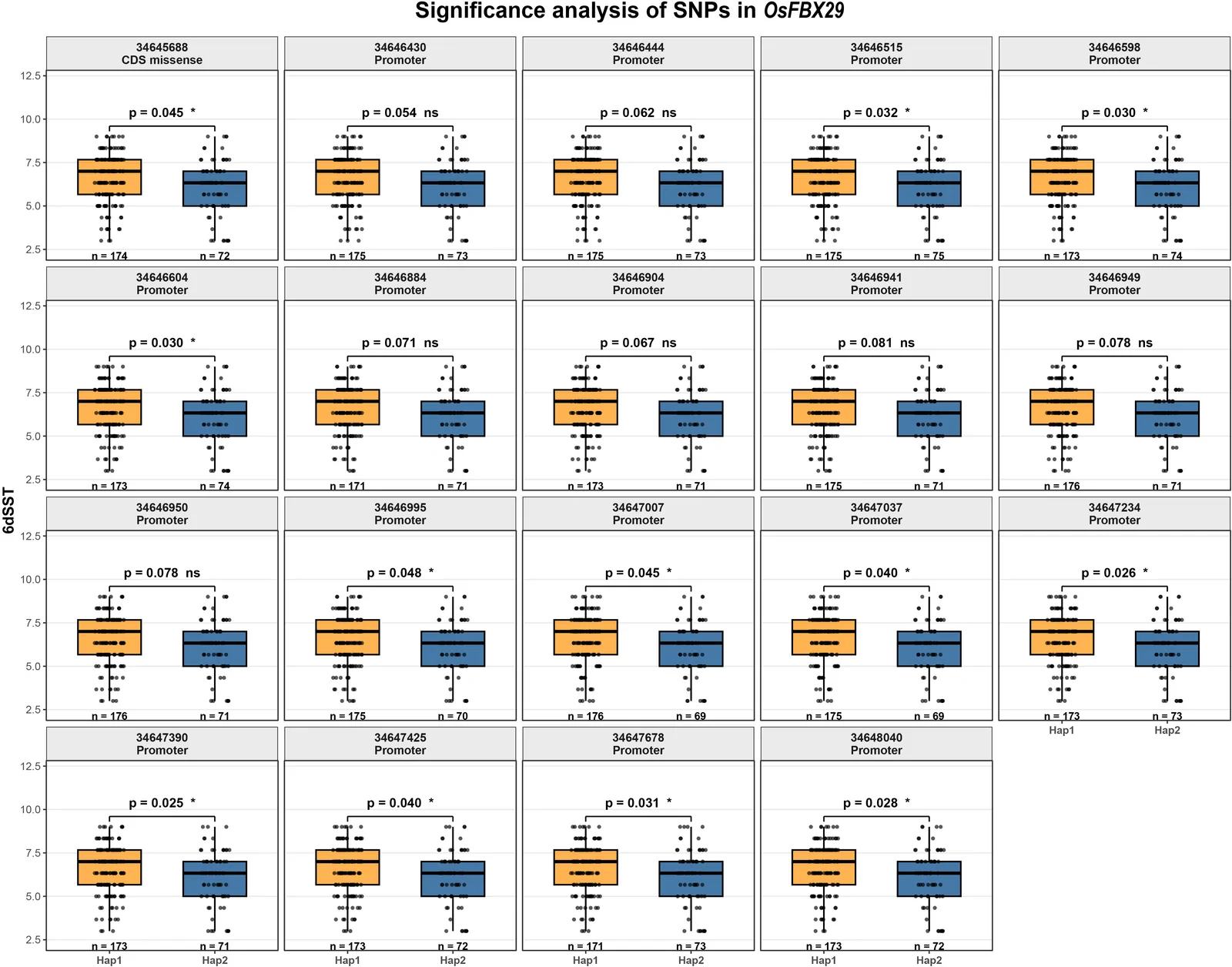
Significance analysis of SNPs in *OsFBX29* for 6dSST. Eighteen SNPs in the promoter and one nonsynonymous SNP in the CDS were analyzed. Hap1 and Hap2 represent the two parental haplotype groups at each site. The number of RILs included in each haplotype group (n) is indicated for each SNP site. Lines with heterozygous or missing genotypes were excluded from the comparison. “ns” indicates no significant difference, whereas * indicates significance at *P* < 0.05.

### Gene co-expression network analysis

RNA-seq was performed on salt-sensitive (S) and salt-tolerant (R) lines at 0 h and 6 h after salt treatment. WGCNA identified seven co-expression modules, among which the turquoise module showed a significant association with salt treatment (**Figure 4**; **Supplementary Figure S6**). This module contained 6,385 genes, including known salt stress-related genes such as *OsRLCK311* (Sade et al., 2020) and *OsMSRA4.1* (Guo et al., 2009). GO enrichment analysis revealed that genes in the turquoise module were predominantly enriched in transcriptional regulation and RNA processing categories, including transcription by RNA polymerase II, regulation of RNA polymerase II-dependent transcription, RNA splicing, and mediator complex, suggesting a role in transcriptional reprogramming during the salt stress response (**Supplementary Figure S7**).

**Figure 4:**
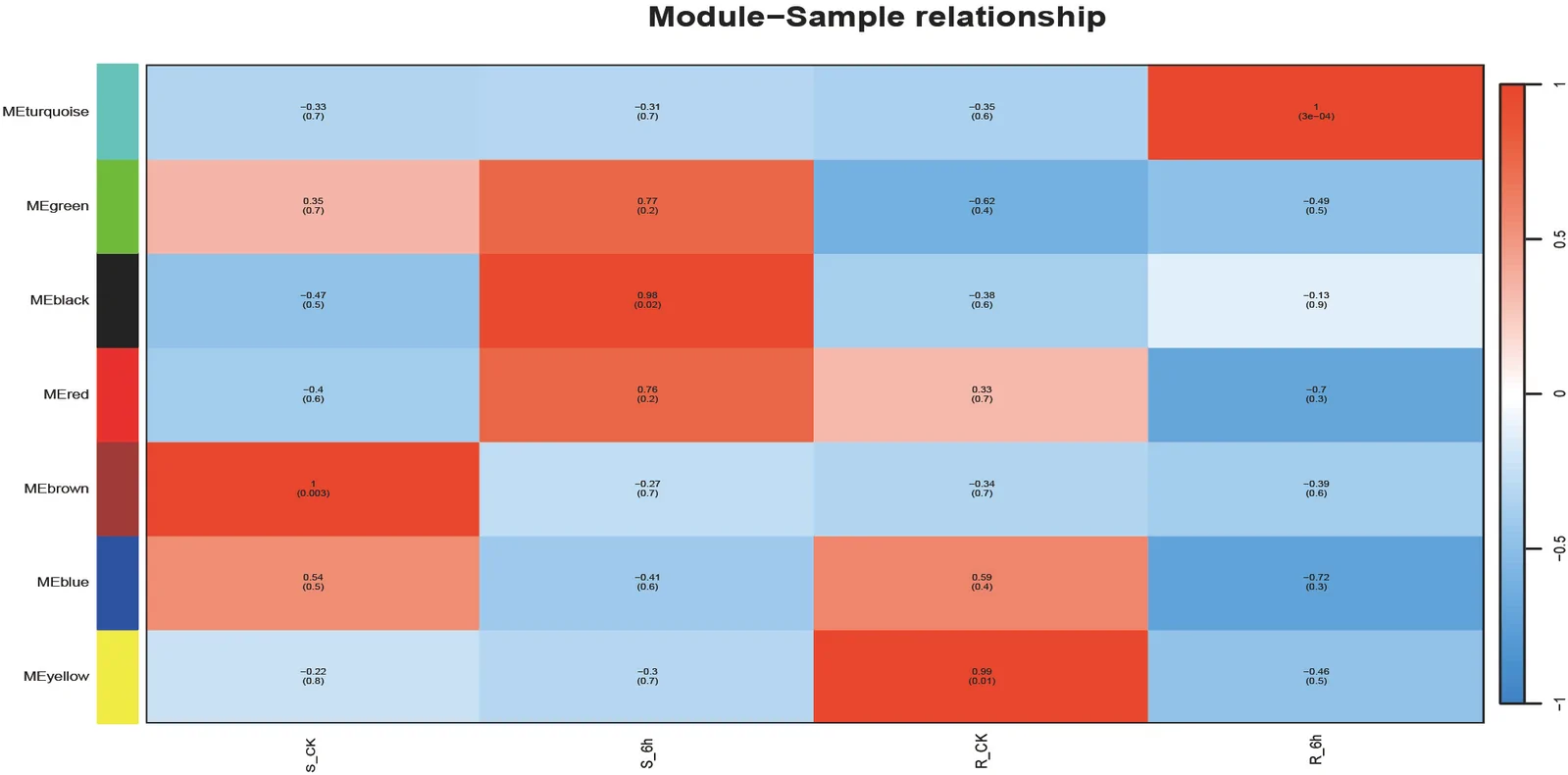
Module–condition relationship analysis based on WGCNA. The heatmap shows correlations between co-expression modules and treatment conditions; each cell displays the Pearson correlation coefficient and corresponding p-value.

WGCNA analysis showed that *OsFBX29*, *OsSWEET6b* (*LOC_Os01g42090*), and *OsRAV9* (*LOC_Os01g04800*) were assigned to the turquoise module. Among these genes, only *OsFBX29* was located within the prioritized QTL regions and was further supported by haplotype analysis. Therefore, *OsFBX29* was prioritized as a strong candidate gene associated with seedling-stage salinity tolerance.

### Expression analysis of *OsFBX29* under salt stress

RNA-seq analysis showed that *OsFBX29* expression increased in the salt-sensitive line, whereas a slight decreasing trend was observed in the salt-tolerant line (**Supplementary Table S3**). To further validate the expression pattern of the prioritized candidate gene *OsFBX29*, qRT-PCR analysis was performed. *OsFBX29* displayed an expression pattern consistent with both the RNA-seq expression trend and the phenotypic contrast between tolerant and sensitive lines. At 6 h after salt treatment, *OsFBX29* expression was significantly downregulated in the salt-tolerant lines but significantly upregulated in the salt-sensitive lines (**Figure 5**). Based on the 6 h/0 h expression ratio, mean *OsFBX29* expression decreased to approximately 0.73-fold in the salt-tolerant lines but increased to approximately 2.15-fold in the salt-sensitive lines relative to 0 h. The relative expression range in the salt-tolerant lines (0.63–0.81) was markedly lower than that in the salt-sensitive lines (1.72–2.76) under salt stress. These results indicate that Hap2-carrying tolerant lines exhibited reduced *OsFBX29* expression under salt stress, whereas Hap1-carrying sensitive lines showed increased expression. This expression pattern is consistent with the haplotype-associated phenotypic differences and provides additional expression-based support for prioritizing *OsFBX29* as a candidate gene associated with seedling-stage salinity tolerance.

**Figure 5:**
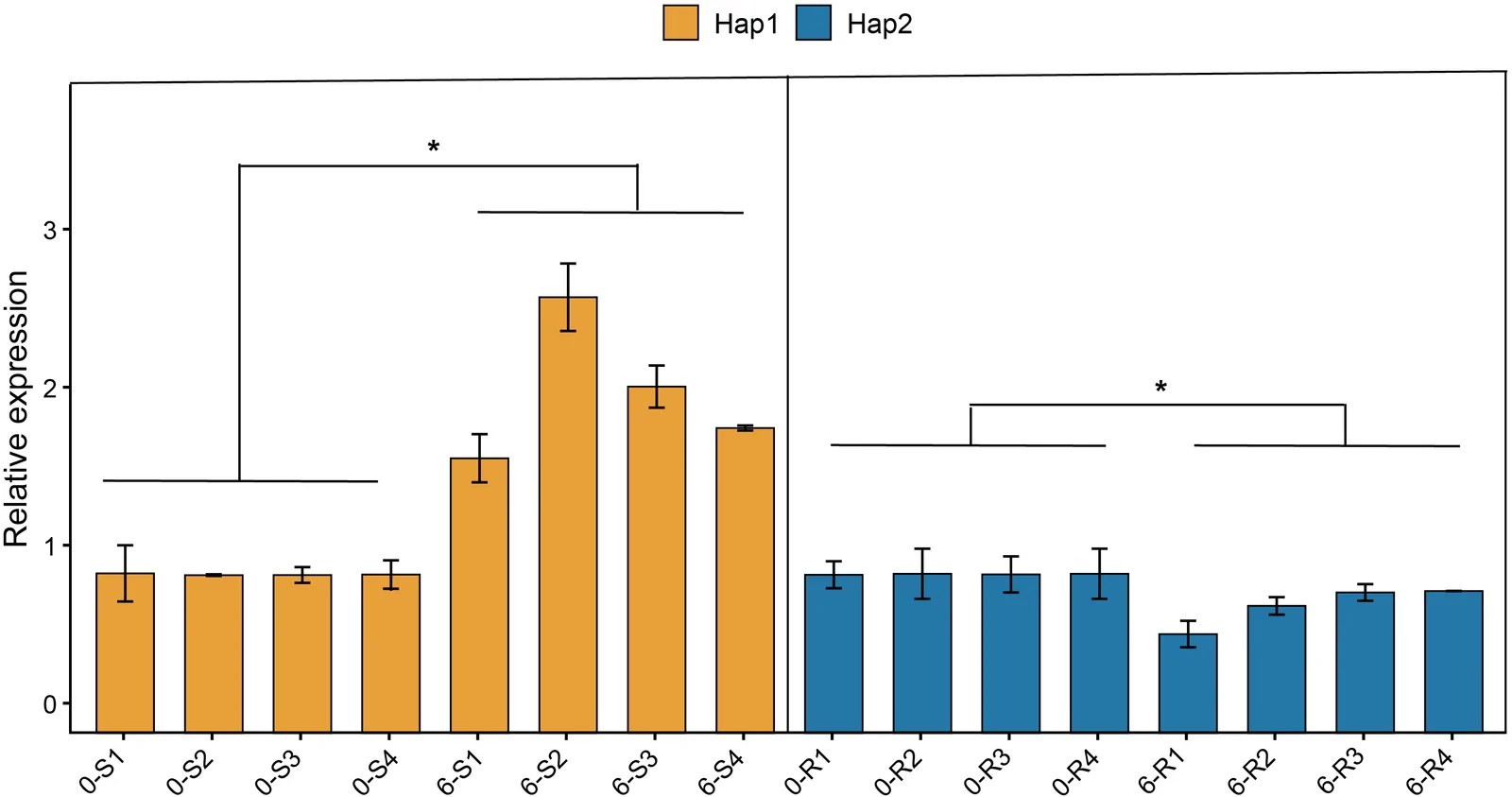
Relative expression levels of *OsFBX29* under salt stress as determined by qRT-PCR. Four Hap1-carrying salt-sensitive lines (S1–S4) and four Hap2-carrying salt-tolerant lines (R1–R4) were used for qRT-PCR analysis. Values are presented as mean ± SD of three biological replicates. “6 h” denotes the 6 h salt-treated group, whereas “0 h” denotes the untreated control. Asterisks indicate significant differences between the 0 h and 6 h groups within Hap1 and Hap2, respectively, as determined by a paired Student’s t-test using the mean expression values of four RIL lines per haplotype (*P* < 0.05).

## Discussion

### Seedling-stage salinity tolerance in rice

Salinity stress is a major environmental constraint affecting rice growth, development, and yield (Hoang et al., 2016; Hussain et al., 2017; Zheng et al., 2023). In recent years, substantial progress has been made in identifying salinity-tolerance-related genes and dissecting the genetic basis of salinity tolerance in rice (Reddy et al., 2017; Chen et al., 2021; Yu et al., 2017; Liu et al., 2019). Because the seedling stage is particularly sensitive to salinity stress, phenotypic evaluation at this stage provides an important basis for elucidating the genetic architecture of salinity tolerance (Tiwari et al., 2022). Previous studies using RIL populations have identified multiple QTLs associated with seedling-stage salinity tolerance, including the *Saltol* locus in IR29 × Pokkali (Thomson et al., 2010), *qST1* and *qST3* in Milyang 23 × Gihobyeo (Lee et al., 2007), and several QTLs in At354 × Bg352 (Dahanayaka et al., 2017). Collectively, these findings indicate that seedling-stage salinity tolerance in rice is a complex quantitative trait governed by multiple loci.

In the present study, the RIL population derived from the cross between the salt-sensitive japonica cultivar JL1 and the salt-tolerant indica cultivar MY23 exhibited substantial phenotypic variation under 0.9% NaCl treatment at the seedling stage. The continuous distribution and clear transgressive segregation of salinity-tolerance-related traits among the RILs indicate that seedling-stage salinity tolerance in this population is a quantitatively inherited trait governed by multiple genetic factors (**Supplementary Figure S1**). This phenotypic variation further suggests that the parental lines carry contrasting alleles for salinity tolerance, supporting the suitability of this population for QTL mapping and candidate-gene identification. In addition, this study evaluated rice salt tolerance under a single salinity level of 0.9% NaCl, which presents certain experimental limitations. The growth responses and physiological regulatory mechanisms of rice germplasm vary significantly under mild, moderate, and severe salt stress conditions. Accordingly, the results of this study are most applicable to moderately saline soils with salt concentrations close to 0.9% NaCl. Three salt-tolerant RIL lines (RIL 69, RIL 143, and RIL 248) were identified based on their consistently favorable performance across the evaluated seedling-stage salinity-tolerance-related traits (**Supplementary Table S4**). However, in highly saline-alkali soils with salt concentrations substantially higher than 0.9% NaCl, the observed tolerance advantages may not be consistently expressed. Future studies will incorporate multiple salinity gradients to establish a more comprehensive stress evaluation system and to identify superior germplasm adapted to different levels of saline-alkali environments.

### Identification and comparison of salinity-tolerance QTLs in rice

In this study, QTL mapping identified 43 QTLs associated with seedling-stage salinity tolerance based on multiple phenotypic indices measured at different time points. To clarify the novelty of these loci, the QTL intervals identified in the present study were compared with previously reported salinity-tolerance-related QTLs and genes based on available physical positions or marker-defined regions anchored to the rice Nipponbare reference genome (Kawahara et al., 2013). Among the 43 QTLs, 23 QTLs (53.49%) showed no clear positional correspondence with previously reported loci and were therefore considered potentially novel. In addition, 11 QTLs each explained more than 10% of the phenotypic variation, suggesting that several loci detected here may have relatively large effects on seedling-stage salinity tolerance. Among the major-effect QTLs, *qISS9* showed the largest effect, explaining 19.78% of the phenotypic variation. Comparison with earlier studies revealed several genomic regions that showed overlap with previously reported salinity-tolerance-related loci. Mohammadi et al. (2013) mapped qPH1.1s on chromosome 1 within the RM246–RM431 interval, corresponding to approximately 27.33–38.89 Mb, which overlapped with *qCH1* and *qSST1*, identified in the present study. Similarly, the *QSkc1* locus reported by Cheng et al. (2012) in the RM297–RM315 region of chromosome 1, corresponding to approximately 32.09–36.73 Mb also overlapped with *qCH1* and *qSST1*. On chromosome 10, the *SalTol10–1* locus reported by Islam et al. (2011) in the RM25092–RM25519 region, corresponding to approximately 5.34–16.79 Mb overlapped with several QTLs identified here, including *qCH10*, *q4dSST10*, *q7dSST10*, *qDSS10*, and *qSST10* within the 12.93–13.14 Mb interval; *qDLR10*, *q5dSST10*, *q6dSST10*, and *qISS10* within the 13.72–13.94 Mb interval; and *q2dSST10* within the 15.74–15.92 Mb interval. In addition, *qSKC10*, previously localized by Qiu et al. (2015) to the RM311–RM467 region on chromosome 10, also overlapped with *qCH10*, *q4dSST10*, *q7dSST10*, *qDSS10*, and *qSST10* identified in the present study.

These overlapping genomic regions provide supporting evidence for the relevance of several genomic regions identified in this study to seedling-stage salinity tolerance, whereas the identification of 23 potentially novel QTLs highlights the added value of the present study and provides additional candidate regions for fine mapping and molecular breeding.

### *OsFBX29* is associated with seedling-stage salinity tolerance

In this study, QTL mapping combined with haplotype analysis, expression profiling, and co-expression network analysis prioritized *OsFBX29* as a strong candidate gene associated with seedling-stage salinity tolerance. Among the haplotypes detected at this locus, Hap2-carrying lines exhibited significantly enhanced salinity tolerance compared with Hap1-carrying lines. qRT-PCR analysis further showed that *OsFBX29* expression was reduced in Hap2-carrying tolerant lines but increased in Hap1-carrying sensitive lines under salt stress, providing additional expression-based support for the association between *OsFBX29* and seedling-stage salinity tolerance.

*OsFBX29* encodes an F-box domain-containing protein and belongs to the large F-box family of ubiquitination regulators in plants (Lechner et al., 2006; Jain et al., 2007). F-box proteins typically function as the substrate-recognition subunits of the SCF (SKP1–CUL1–F-box) E3 ubiquitin ligase complex, recruiting specific target proteins and promoting their degradation via the 26S proteasome pathway. Through this mechanism, F-box proteins play important roles in hormone signaling and abiotic stress responses (Lechner et al., 2006; Fan et al., 2021; Xu et al., 2014; Yan et al., 2011). *OsFBX257* has been reported to enhance salt tolerance in transgenic rice and Arabidopsis, whereas *OsFBX148* enhances salinity tolerance when heterologously overexpressed in Arabidopsis, possibly by regulating Na^+^ distribution and maintaining ROS homeostasis (Xu et al., 2014; Fan et al., 2021). Given its F-box domain and the reported roles of salt-related F-box proteins, *OsFBX29* may be involved in salt-stress regulation through ubiquitin-mediated protein turnover, providing a plausible biological context for its association with salinity tolerance. Notably, the reduced expression of *OsFBX29* observed in Hap2-carrying tolerant lines further supports its potential association with seedling-stage salinity tolerance.

In summary, phenotypic and expression analyses supported a potential association between variation at the *OsFBX29* locus and seedling-stage salinity tolerance, with lower *OsFBX29* expression observed in Hap2-carrying tolerant lines under salt stress.

## Conclusion

In this study, we identified 43 QTLs associated with seedling-stage salinity tolerance in rice. Through integrated analyses encompassing gene annotation within QTL intervals, haplotype analysis, co-expression network analysis, and qRT-PCR validation, *OsFBX29* was prioritized as a promising candidate gene associated with seedling-stage salinity tolerance. Nevertheless, its biological function and causal role in salinity tolerance require further functional validation through genetic approaches in future studies. Our findings provide new insights into the genetic basis of seedling-stage salinity tolerance and offer useful candidate regions and genes for future fine mapping, functional characterization, and salinity-tolerance breeding.

## Data Availability

The raw genome sequence data reported in this paper have been deposited in the National Genomics Data Center (NGDC, part of the China National Center for Bioinformation (CNCB), under accession codes CRA008901 (https://ngdc.cncb.ac.cn/gsa/search?searchTerm=CRA008901) and CRA050344 (https://ngdc.cncb.ac.cn/gsa/browse/CRA050344). The transcriptome sequencing data generated in this study have been submitted to the National Genomics Data Center (NGDC) under BioProject accession number PRJCA067271 (https://ngdc.cncb.ac.cn/search/specific?db=bioproject&q=PRJCA067271).

## Supplementary material

Supplementary Figure 1 Phenotypic distributions of salinity-tolerance-related traits in the RIL population. **(A–F)** Salinity tolerance scores from day 2 to day 7 after salt treatment (2dSST–7dSST); **(G)** seedling survival rate (SSR); **(H)** chlorophyll content (CH); **(I)** dead leaf rate (DLR); **(J)** DLR-derived salinity tolerance score (DSS); **(K)** salinity index (SI); **(L)** SI-derived salinity tolerance score (ISS); and **(M)** single-plant salinity tolerance score (SST). Red arrows indicate the phenotypic values of P1 (JL1), and green arrows indicate those of P2 (MY23).

Supplementary Figure 2 Correlation analysis of salinity-tolerance-related traits in the RIL population. Numbers indicate Pearson correlation coefficients. Red and blue indicate positive and negative correlations, respectively. *indicates significance at *p* ≤ 0.05, and **indicates significance at *p* ≤ 0.01.

Supplementary Figure 3 Significance analysis of SNPs in *OsFBX339* for 6dSST. Hap1 and Hap2 represent RILs carrying the homozygous JL1-derived and MY23-derived alleles at each SNP site, respectively. “ns” indicates no significant difference, whereas *indicates significance at *P* < 0.05.

Supplementary Figure 4 Significance analysis of SNPs in *OsMYB21* for 6dSST. Hap1 and Hap2 represent RILs carrying the homozygous JL1-derived and MY23-derived alleles at each SNP site, respectively. “ns” indicates no significant difference, whereas * indicates significance at *P* < 0.05.

Supplementary Figure 5 Significance analysis of SNPs in *OsSub59* for 6dSST. Hap1 and Hap2 represent RILs carrying the homozygous JL1-derived and MY23-derived alleles at each SNP site, respectively. “ns” indicates no significant difference, whereas * indicates significance at *P* < 0.05.

Supplementary Figure 6 Weighted gene co-expression network analysis (WGCNA) of RNA-seq data. **(A)** Soft-thresholding power determination for network construction. **(B)** Hierarchical clustering dendrogram of co-expression modules identified by WGCNA; module colors indicate distinct co-expression modules.

Supplementary Figure 7 GO enrichment analysis of genes in the turquoise co-expression module.
